# Exercise training alone or in combination with high-protein diet in patients with late onset Pompe disease: results of a cross over study

**DOI:** 10.1186/s13023-020-01416-6

**Published:** 2020-06-06

**Authors:** Annalisa Sechi, Lucrezia Zuccarelli, Bruno Grassi, Rita Frangiamore, Ramona De Amicis, Mauro Marzorati, Simone Porcelli, Annarita Tullio, Anna Bacco, Simona Bertoli, Andrea Dardis, Lea Biasutti, Maria Barbara Pasanisi, Grazia Devigili, Bruno Bembi

**Affiliations:** 1 Regional Coordinator Center for Rare Diseases, Academic hospital of Udine, p.zzale SM della Misericordia 15, 33100 Udine, Italy; 2 Department of Medicine, University of Udine, Udine, Italy; 3 Neuroimmunology and Muscle Pathology Unit, Fondazione IRCCS Istituto Neurologico Carlo Besta, Milan, Italy; 4 International Center for the Assessment of Nutritional Status (ICANS), Department of Food Environmental and Nutritional Sciences (DeFENS), University of Milan, Milan, Italy; 5 Institute of Biomedical Technologies, National Research Council, Segrate, Italy; 6 Institute of Hygiene and Clinical Epidemiology, Academic hospital of Udine, Udine, Italy; 7 Division of Endocrinology, Metabolic Diseases and Nutrition, Academic Hospital of Udine, Udine, Italy; 8 Neurological Unit 1, Fondazione IRCCS Istituto Neurologico Carlo Besta, Milan, Italy

**Keywords:** Pompe disease, High-protein diet, Enzyme replacement therapy, Exercise training, Exercise tolerance

## Abstract

**Background:**

Late onset Pompe disease (LOPD) is a lysosomal neuromuscular disorder which can progressively impair the patients’ exercise tolerance, motor and respiratory functions, and quality of life. The available enzyme replacement therapy (ERT) does not completely counteract disease progression. We investigated the effect of exercise training alone, or associated with a high-protein diet, on the exercise tolerance, muscle and pulmonary functions, and quality of life of LOPD patients on long term ERT.

**Methods:**

The patients were asked to participate to a crossover randomized study comprehending a *control* period (free diet, no exercise) followed by 2 intervention periods: *exercise* or *exercise + diet*, each lasting 26 weeks and separated by 13 weeks washout periods. Exercise training included moderate-intensity aerobic exercise on a cycle ergometer, stretching and balance exercises, strength training. The diet was composed by 25–30% protein, 30–35% carbohydrate and 35–40% fat. Before and after each period patients were assessed for: exercise tolerance test on a cycle-ergometer, serum muscle enzymes, pulmonary function tests and SF36 questionnaire for quality of life. Compliance was evaluated by training and dietary diaries. Patients were contacted weekly by researchers to optimize adherence to treatments.

**Results:**

Thirteen LOPD patients, median age 49 ± 11 years, under chronic ERT (median 6.0 ± 4.0 years) were recruited. Peak aerobic power (peak pulmonary O_2_ uptake) decreased after *control*, whereas it increased after *exercise,* and more markedlyafter *exercise + diet*. Serum levels of lactate dehydrogenase (LDH) significantly decreased after *exercise + diet;* both creatine kinase (CK) and LDH levels were significantly reduced after *exercise + diet* compared to *exercise.* Pulmonary function showed no changes after *control* and *exercise*, whereas a significant improvement of forced expiratory volume in 1 sec (FEV1) was observed after *exercise + diet*. SF36 showed a slight improvement in the “mental component” scale after *exercise*, and a significant improvement in “general health” and “vitality” after *exercise + diet*. The compliance to prescriptions was higher than 70% for both *diet* and *exercise*.

**Conclusions:**

Exercise tolerance (as evaluated by peak aerobic power) showed a tendency to decrease in LOPD patients on long term ERT. Exercise training, particularly if combined with high-protein diet, could reverse this decrease and result in an improvement, which was accompanied by improved quality of life. The association of the two lifestyle interventions resulted also in a reduction of muscle enzyme levels and improved pulmonary function.

## Introduction

Pompe disease, or glycogen storage disease type II, is a rare inherited metabolic disorder due to the deficiency of the enzyme acid alpha glucosidase (GAA), which normally breaks down the glycogen inside the lysosomes of various cellular types. The GAA deficiency results into glycogen accumulation, especially in fibers of skeletal, respiratory and cardiac muscles; the consequence is a progressive invalidating disease [1]. In clinical practice Pompe disease has been differentiated in two main subtypes: the classic infantile form, rapidly progressive with cardiac involvement, and the late-onset form (LOPD), characterized by a slower disease progression and free from cardiac involvement [2]. In the caucasian population the estimated incidence is 1/138.000 for the infantile form and 1/57.000 for LOPD [3]. Exercise intolerance, defined as the inability to produce/maintain adequate force or power to accomplish a task, is a key clinical manifestation of LOPD. Exercise intolerance appears early in the natural history of the disease and significantly impairs the patients’ clinical picture and quality of life [4–6]. The specific enzyme replacement therapy (ERT) with recombinant alpha-glucosidase became available in 2006 and showed to be effective on motor and respiratory functions of LOPD patients [7, 8], leading to an improved exercise tolerance [5]. However, different authors observed that the positive effects of ERT, evident in the first years of treatment, seem to stabilize or even decline afterwards [9, 10]. Due to the limitations of ERT, the development and implementation of ancillary therapeutic approaches are important in optimizing the care of Pompe patients.

Before the introduction of ERT, the effects of high-protein diet associated to exercise training in LOPD have been studied by Slonim et al. [11]; the authors demonstrated a slowing in the disease progression rate [11]. In the post-ERT era, however, these life-style interventions were mostly disregarded in clinical practice. Recently, two studies demonstrated beneficial effects of aerobic exercise in LOPD patients treated with ERT [12, 13]. To the best of our knowledge, however, no studies have been conducted on the association of exercise and a high-protein diet in these patients.

Since the high-protein diet and exercise would act on different mechanisms, we hypothesized that the combined effects of the two interventions could be synergic. Thus, the primary aim of the present study was to evaluate the effects of an individualized exercise training program, alone or in association with a high-protein diet, on exercise tolerance, muscle and pulmonary functions, and quality of life in LOPD patients chronically treated with ERT. The secondary aim was to evaluate the patients’ compliance to the proposed intensive life-style interventions, and therefore their feasibility in clinical practice.

## Patients and methods

Patients were enrolled by two different centres: the Regional Coordinator Centre for Rare Disease of the Academic Hospital of Udine, and the Carlo Besta Neurological Institute of Milan. Main inclusion criteria were: age ≥ 18 years; diagnosis of LOPD confirmed by enzymatic test and/or genetic analysis; regular ERT for at least 2 years. Main exclusion criteria were: presence of significant cardiovascular diseases (assessed by electrocardiogram and echocardiography); wheelchair bound; 24 h ventilator support; pregnancy. Patients who already performed regular physical activity or followed specific diets were also excluded.

### Study protocol

The study was designed as a partially blinded, randomized, crossover study. Blinding was applied to the researchers working at the laboratory assays, instrumental testing and data analysis. Following a *control* period (free diet, no exercise training), participants were randomly assigned to a sequence of two treatment periods: *exercise* alone and *exercise + diet*Each period lasted 26 weeks and was followed by a 13-week wash-out (free diet, no exercise training). Patients continued ERT during the entire study period, at the usual dosage (20 mg/kg every 2 weeks).

The study was approved by the ethical committees of the Friuli Venezia Giulia region and of the Carlo Besta Neurological Institute of Milan. All patients signed an informed consent before entering the study.

### Exercise training

Exercise training was prescribed after an initial evaluation of exercise tolerance and muscular strength carried out in the exercise physiology laboratories (Department of Medicine, University of Udine, Institute of Bioimaging and Molecular Physiology, National Research Council, Milan) participating to the study. Patients were carefully instructed about the training protocol during the initial visits at the exercise physiology laboratories. Training, conducted at home, was personalized for each patient and comprehended 4 sessions/week, 1 h/session. Each session included: 1) Warm-up exercises. 2) Stretching and balance exercises (10–15 min). 3) Strenght training at very moderate loads involving the main muscle groups of the upper (shoulder adductions and abductions in the frontal plane, bicep curls in the sagittal plane, chest press in the sagittal plane and back rows in the sagittal plane), lower limbs (leg adductions and abductions in the frontal plane and back squats) and of the trunk (spinal rotations in the transverse plane), carried out by utilizing elastic bands (THERA-BAND, Akron, Ohio, USA) (15 min). During the initial evaluation of the patients the length of the elastic band was set corresponding to a “very moderate” resistance, according to the manufacturer’s instructions. Three series of 10 repetitions were performed for each movement. The resistance of the bands and the number of repetitions were kept constant throughout the study. 4) Moderate-intensity aerobic exercise on a cycle ergometer, carried out at about 60% of the patient’s maximal heart rate (HR) determined during the preliminary incremental test (see below) (30–40 min). A cycle ergometer with a HR-meter (Ergo bike, Cardio Pro, Daum Electronic, Germany) was shipped to each patient’s domicile for the training periods. The HR-meter allowed the downloading of data on a personal computer. Since aerobic training intensity was targeted to a specific HR value, and not to a specific work rate, the adjustment of training intensity to increases or decreases of exercise tolerance was automatic, without the need for *interim* evaluations with incremental exercise tests during each period of the study.

### Dietary treatment

Before receiving the dietary prescription, the patients underwent a complete nutritional and dietary assessment at the Academic Hospital of Udine or at the International Center for the Assessment of Nutritional Status (ICANS), University of Milan.

The patients were asked to follow a personalized high-protein diet, composed by 25–30% protein, 30–35% carbohydrate and 35–40% fat. Caloric requirements were calculated by sex, age, body weight and height according to the Harris-Benedict formula [14], sedentary physical activity level (PAL = 1,45 (SINU-Società Italiana di Nutrizione Umana. LARN- revision 2014), and nutritional status at baseline.

Daily protein amount was distributed in 6 meals: 20% at breakfast, 25% at lunch, 25% at dinner and the remaining 30% in three snacks (10% during the morning, 10% during the afternoon and 10% in the last snack after dinner). A neutral or flavored powder was used to create ready-to-drink products rich in protein for breakfast in patients who did not like salty breakfast. Only dietary protein sources were used in other meals (animal proteins 49 ± 11%, vegetable proteins 27 ± 9%).

### Efficacy assessments

The following assessments were performed before and after each intervention period (*control* period included).

### Anthropometrics and body composition measurements

The anthropometric and body composition measurements included weight, height, body mass index (BMI, kg/m^2^), waist circumference.

#### Bioelectrical impedance (BIA)

Total body water, intracellular water, and extracellular water (ECW, L) were measured by the tetrapolar 8-point tactile electrode system using an impedance (InBody S10, Biospace, Seoul, Korea) at 1, 5, 50, 250, 500, and 1000 kHz. BIA was performed with patients fasting for at least 4 h, without having exercised in the last 12 h, without having drunk water in the last hour.

### Functional evaluation of exercise tolerance

Experiments were conducted under medical supervision, and patients were continuously monitored by 12-lead electrocardiography (ECG). A mechanically braked cycle ergometer (Monark Ergomedic 839E) was utilized. Pedalling frequency was digitally displayed to the subjects, who were asked to keep a constant cadence throughout the test, between 60 and 70 rpm. Each patient had chosen his preferred cadence during practice trials, and this cadence was maintained during each repetition. Patients were allowed time to gain familiarity with the researchers and the experimental set-up, were carefully instructed about the procedures and were familiarized with the protocol using short practice runs before starting the study.

A specific functional evaluation protocol was utilized as previously described [6]. The patients performed a low-intensity constant work rate (CWR) exercise for 8 min or until voluntary exhaustion, identified as the inability to maintain the desired cadence, despite verbal encouragement. In the patients who completed the CWR exercise, the test was immediately followed by an incremental exercise up to voluntary exhaustion. During the incremental exercise, work rate was increased by 5–10 W every minute. Pulmonary ventilation (V̇E), tidal volume (VT), respiratory frequency (fR), O_2_ uptake (V̇O_2_) and CO_2_ output (V̇CO_2_) were determined on a breath-by-breath basis by means of a metabolic unit (Quark b^2^, Cosmed, Italy). Expiratory flow measurements were performed by a turbine flow meter calibrated before each experiment by a 3 L syringe at three different flow rates. Calibration of O_2_ and CO_2_ analysers was performed before each experiment by utilizing gas mixtures of known composition. The gas exchange ratio (R) was calculated as V̇CO_2_/ V̇O_2_. Arterial oxygen saturation (SpO_2_) was determined at the earlobe by pulse-oximetry (Oximeter Xpod, 3012LP, Cosmed, Italy).

Heart rate was determined by ECG.

For all variable’s values determined at voluntary exhaustion were considered “peak” values. The time to exhaustion was also determined.

### Other parameters of clinical outcome

Muscle force of the right lower limb (knee angle at 110°) and right upper limb (elbow angle at 90°) were measured during an isometric maximal voluntary contraction (MVC), during both flexion and extension, using a TSD121C isometric dynamometer (Biopac Systems Inc., Santa Barbara, CA, USA). Force analog output was sampled at a frequency of 1 kHz, and was acquired with the BIOPAC System MP100 (Biopac Systems Inc., Santa Barbara, CA, USA) using AcqKnowledge software version 3.7.2. Peak values of every trial were identified and retained for data analysis.

Serum muscular enzymes (creatinekinase CK, lactate dehydrogenase LDH, alanine transaminase ALT, and aspartate transaminase AST), and other hematologic parameters (glucose, hemoglobin, lipid profile, albumine, creatinine) were measured by standard procedures.

Pulmonary function tests included a standard spirometry with the evaluation of the vital capacity (VC) and the forced expiratory volume in 1 sec (FEV_1_), reported as % of normal values [15].

Walking distance during the 6-min walking test (6MWT) was evaluated according to the American Thoracic Society guidelines [16].

Modified Walton Scale scores were assessed according to Slonim et al. 2006 [17].

### Quality of life

Quality of life was assessed using the validated, standardized Italian version of the SF36 questionnaire [18]. The 36 items were divided into eight concepts plus one health comparison question. The eight concepts to be scored were: Physical Functioning, Role-Physical, RoleEmotional, Social Functioning, Bodily Pain, Mental Health, Vitality, General Health Perceptions, and Change in Health. The raw scale scores were computed by summing across items within each concept. The raw scores were transformed to a 0–100 scale using the formula = [(actual raw score - lowest possible raw score)/ possible raw score rangel • 100] and then normalized to a Z-score. The physical and mental component summary measures were also computed and transformed to norm-based scores (mean 50, standard deviation 10) using the mean, standard deviation, and scoring coefficients from the US general population [19]. Both for combined scales and summary measures, higher scores indicate higher levels of physical functioning or psychological well-being.

### Compliance assessment

Patients’ compliance to the exercise training was assessed by analysis of the 7 days training diaries completed during the interventions period, and by the analysis of the data of the HR-meter of the cycle-ergometers. Three-days weighted-dietary diaries, completed every month during all the study periods, were used to evaluate food intake, and to assess dietary habits. Each day of the food record was assessed for completeness by a trained dietician, and then coded and analyzed using the WinFood® 3.0 software. During the intervention periods weekly phone-calls or e-mails were utilized by the research staff to optimize adherence to treatments.

### Statistical analysis

Descriptive statistics were performed on categorical and numerical variables. Frequency distributions were used for categorical variables. For numerical variables we considered median, interquartile range, minimum and maximum values. Due to the low sample size, analyses were conducted using non parametric tests. After checking for the absence of a carry-over effect (two-stage Grizzle model and graphical assessment), pre and post intervention results were compared by Wilcoxon rank sum test for paired data.

Exercise and exercise+diet period were also compared by applying the Wilcoxon signed rank sum test for paired data to the difference between the two periods for the percentage before - after difference data.

All statistical analyses were performed using SAS© software, version 9.4 (SAS institute, Inc., Cary, NC, USA) and R software, version 3.4.2. The significance level was set at *p* < 0.05. In some specified cases, considering the relatively small population included in the study, data of interest with a *p*-value < 0.10 were reported.

## Results

Thirteen LOPD patients were enrolled, two couples were brothers (UD02- UD04; MI05-MI06), whereas the other patients were unrelated. The patients general characteristics at study entry are summarized in Table 1; median age at the beginning of the study was 49.0 ± 11.0 years, all were on long term ERT (median treatment duration 6.0 ± 4.0 years). The disease severity was variable, and Walton Scale scores ranged from 0 (all activities normal) to 6 (walking only with callipers); 8 patients were on non-invasive nocturnal ventilation (NIV). Regarding genotype, all participants but one (patient UD07) were compound heterozygous for the common LOPD mutation c.-32-13 T > G.

**Table 1:** General characteristics of patients

Patient ID	Gender	Age at study entry	Age at diagnosis	Years on ERT	Walton scale score	BMI before diet	Ventilatory support
UD01	M	19	3	9	0	20.7	no
UD02	M	48	41	6	0	30.5	no
UD03	F	49	29	6	3	17.5	no
UD04	M	55	45	9	3	NA	NIV
UD05	F	46	25	9	6	NA	NIV
UD06	F	42	29	9	3	23.7	NIV
UD07	F	45	16	10	6	16.4	NIV
MI01	F	69	66	3	3	21	no
MI02	M	71	67	4	3	19.6	NIV
MI03	F	36	32	5	3	27.8	no
MI04	F	56	37	6	3	NA	NIV
MI05	F	59	54	5	3	28.4	NIV
MI06	M	51	45	6	2	22.3	NIV
Median ± IQR		49.0 ± 11.0	37.0 ± 16.0	6.0 ± 4.0		21.65 ± 6.9	

*NIV* nocturnal non invasive ventilation

*NA* not applicable

Two patients (UD04 and MI04) dropped out of the study, for personal reasons, after the *control* period, another one (UD05) completed the *control* and the *exercise* periods but not the *exercise + diet* period. Four patients (UD06, UD07, MI05, MI06) were enrolled late during the study, and could not perform the *control* period. Therefore, data before and after the *control* period were available for 9 patients, data before and after *exercise* were available for 11 patients, and data before and after *exercise + diet* were available for 10 patients.

Three out of 10 patients who completed the *exercise + diet* period were overweight at baseline (UD02, MI03, MI05), and therefore received a hypocaloric diet with a caloric deficit corresponding to 20% of their daily energy expenditure, whereas the other patients received a normocaloric diet. The exercise and diet interventions, performed at home, were in general well tolerated.

The two-stage Grizzle model and the graphical assessment excluded a carry-over effect between the intervention periods. Comparison of parameters obtained before and after each period showed the following results:

### Anthropometrics and body composition measurements

When considering all patients, BMI (kg/m^2^) significantly decreased after the *exercise + diet* period (*p* < 0.05), going from a median value of 21.7 ± 6.9 before to 21.3 ± 5.7 after the intervention. No differences occurred in body composition, as measured by bioelectrical impedance, across the study.

### Functional evaluation of exercise tolerance

Peak values of the investigated variables are presented in Table 2. Both when expressed as L^.^min^− 1^ and after the normalization for body mass (mL^.^kg^-1.^min^− 1^) V̇O_2_ peak values (maximal aerobic power) decreased after *control* (*p* = 0.004), whereas they increased after *exercise* (*p* = 0.05) and showed a more significant improvement after *exercise + diet* (*p* = 0.009). Also peak work rate and the time to exhaustion presented similar patterns of change compared to those observed for V̇O_2_ peak, although a statistically significant difference was not always reached, presumably as a consequence of data scattering. No arterial desaturation was observed in any of the experimental conditions.

**Table 2:** Peak values of the main variables determined during incremental exercise

	Control			Exercise			Exercise + Diet		
	Before	After	***p***-value	Before	After	***p***-value	Before	After	***p***-value
**VO2 peak (ml/min/kg)**	17,77 ± 6,96	15,88 ± 4,78	**0,039**	19,24 ± 18,39	21,57 ± 13,26	0,053	20,22 ± 7,27	22,22 ± 4,56	**0,009**
**VO2 peak (l/min)**	1,19 ± 1,43	1,16 ± 0,77	**0,023**	1,23 ± 1,16	1,37 ± 0,62	**0,019**	1,21 ± 0,56	1,38 ± 0,44	**0,027**
**HR peak (bpm)**	143 ± 18	130 ± 14	0,16	137 ± 41	143 ± 37	0,865	146,5 ± 30	148 ± 44	0,113
**HR peak (% pred)**	85 ± 2	70 ± 20	**0,042**	82 ± 25,5	80 ± 14,5	0,905	84,5 ± 5,75	90 ± 16,25	0,059
**Work rate peak (watt)**	55 ± 35	45 ± 40	0,375	75 ± 80	75 ± 70	**0,023**	62,5 ± 55	72,5 ± 55	**0,093**
**VCO2 peak (l/min)**	1,37 ± 0,89	1,31 ± 0,95	0,195	1,34 ± 1,25	1,46 ± 0,93	0,637	1,52 ± 0,60	1,64 ± 0,76	0,039
**VE peak (l/min)**	45,02 ± 25,14	40,59 ± 27,39	0,195	42,48 ± 45,77	41,62 ± 36,56	0,464	47,98 ± 20,42	49,62 ± 24,29	0,064
**R peak**	1,06 ± 0,1	1,07 ± 0,12	0,234	1,1 ± 0,12	1 ± 0,16	0,224	1,08 ± 0,21	1,13 ± 0,16	0,75
**time to exhaustion (min)**	14 ± 8	13 ± 4	0,142	15 ± 15	16 ± 8	**0,014**	13 ± 6,25	16,5 ± 4,5	0,124

Data are expressed as median ± interquartile range before and after each intervention period

*VO2* oxygen uptake, *HR* heart rate, *VCO2* CO2 output, *VE* pulmonary ventilation, *R* gas exchange ratio

No significant differences in the peak values of ventilatory variables (V̇E, VT, fR, V̇CO_2_) were observed across conditions. The same applies to R peak values. In all conditions mean R peak values were higher than 1.0, suggesting that the exercise was indeed, on average, substantially maximal for the patients. HR peak values were, in all conditions, substantially lower than the age-predicted “normal” values, suggesting that the exercise, although exhausting (see R peak) did not represent a maximal burden for the cardiovascular system, thereby suggesting a mainly “peripheral” (locomotor muscles) limitation to exercise tolerance.

### Other parameters of clinical outcome

Results of this section are presented in Table 3.

**Table 3:** Other parameters of clinical outcome

	Control			Exercise			Exercise + Diet		
	Before	After	***p***-value	Before	After	***p***-value	Before	After	***p***-value
**Arms Extensors (N)**	105,13 ± 46,27	107 ± 103,21	0,8125	46,11 ± 84,42	55,36 ± 76,91	0,3125	96,86 ± 96,02	87,4 ± 101,1	0,4609
**Arms Flexors (N)**	104,48 ± 106,84	123,38 ± 80,9	0,5469	74,8 ± 79,88	64,74 ± 59,54	0,25	97,65 ± 79,4	89,03 ± 78,78	0,1953
**Legs Extensors (N)**	164,23 ± 111,47	185,95 ± 120,07	0,5469	136,62 ± 186,13	148,5 ± 81,51	0,3125	179 ± 199,61	152,86 ± 248,1	0,9453
**Legs Flexors (N)**	120,87 ± 85,22	94,88 ± 74,49	0,9375	80,62 ± 79,91	55,5 ± 47,26	0,2109	67,7 ± 33,84	79,73 ± 60,5	0,3125
**CK (U/L)**	280 ± 257	279 ± 284	0,2383	258 ± 262	371 ± 213	0,4131	272,5 ± 218	255 ± 104	0,1406
**LDH (U/L)**	322 ± 209	325 ± 215	0,1367	320 ± 218	320 ± 257	0,2012	303 ± 278	261,5 ± 240	**0,0469**
**6MWT (m)**	404 ± 93	412 ± 127	0,4258	425 ± 151	420 ± 152	0,166	416,8 ± 129	409 ± 119,2	1
**VC (%)**	70 ± 33	71 ± 31	0,1797	71 ± 42	71 ± 48,5	0,9844	75 ± 42	89,5 ± 44	0,0625
**FEV1 (%)**	70 ± 33	71 ± 38	0,3789	71 ± 42	72,4 ± 39,5	0,4844	66,5 ± 53	85 ± 49	**0,0391**

Data are expressed as median ± interquartile range before and after each intervention period

*6MWT* maximum walking distance measured by the 6 min walking test

*VC* vital capacity, *FEV1* forced expiratory volume in 1 s

No significant differences were found in muscle strength, during both flexion and extension exercises of the lower and upper limbs, in after vs. before all experimental conditions.

Serum muscle enzymes showed a significant decrease of LDH after the *exercise + diet* period.

Comparing *exercise* and *exercise + diet* periods by the Wilcoxon signed rank sum test for paired data, statistically significant differences were found for CK and LDH between the two periods for the % difference (before - after), that corresponds to a significant reduction in CK and LDH levels (*p* = 0.03 and *p* = 0.04) after the *exercise + diet* period, compared to the after *exercise* period data.

No differences were found in blood glucose, hemoglobin, lipid profile, albumin, creatinine levels throughout the study.

No significant differences were found for the distance walked during the 6MWT.

The Walton scale score of each patient did not change during the study.

Pulmonary function tests showed a trend toward improvement in VC% (*p* = 0.06) and a significant increase in FEV_1_% (*p* = 0.04) after the *exercise + diet* period, whereas no changes were observed for these variables in the other experimental conditions.

### Quality of life

No differences were found after the *control* period, while after the *exercise* period there was a trend toward improvement in the *mental component scale* (*p* = 0.07), and after the *exercise + diet* period improvements in several thematic areas were described: *mental health* (*p* = 0.07), *mental component scale* (*p* = 0.09); the observed differences reached statistical significance in *general health* (*p* = 0.03), and *vitality* (*p* = 0.03) (Table 4).

**Table 4:** Quality of life before and after *exercise* and *exercise + diet* periods

	Exercise			Exercise + Diet		
	Median score Before	Median Intra personal variation	***p***-value	Median score Before	Median Intra personal variation	***p***-value
**PF**	25	0.00	0.812	45	5.00	0.375
**RP**	50	0.00	0.406	25	0.00	0.375
**BP**	52	0.00	0.562	52	0.00	0.437
**GH**	45	−5.00	0.578	30	5.00	**0.031**
**VT**	40	0.00	0.25	40	10.00	**0.031**
**SF**	75	12.50	0.156	50	0.00	0.375
**RE**	100	0.00	0.75	66.67	0.00	0.625
**MH**	72	4.00	0.152	56	12.00	0.070
**PCS**	34.25	0.26	0.652	33.61	1.83	0.25
**MCS**	53.92	3.06	0.074	41.15	4.29	0.097

*PF* Physical Functioning, *RP* Role-Physical, *BP* Bodily Pain, *GH* General Health, *VT* Vitality, *SF* Social Functioning, *RE* Role Emotional, *MH* Mental Health, *PCS* Physical Component Scale, *MCS* Mental Component Scale

### Compliance

From the analysis of the training diaries and the HR-meters of the cycle-ergometers, the level of adherence to exercise prescriptions were elevated: for the warm up period the percentage of adherence to the prescribed exercise sessions was 69%, for strength training 61%, for cycling 74%, for stretching 69%. These percentages were not significantly different between the *exercise* and the *exercise + diet* periods.

**Fig. 1:**
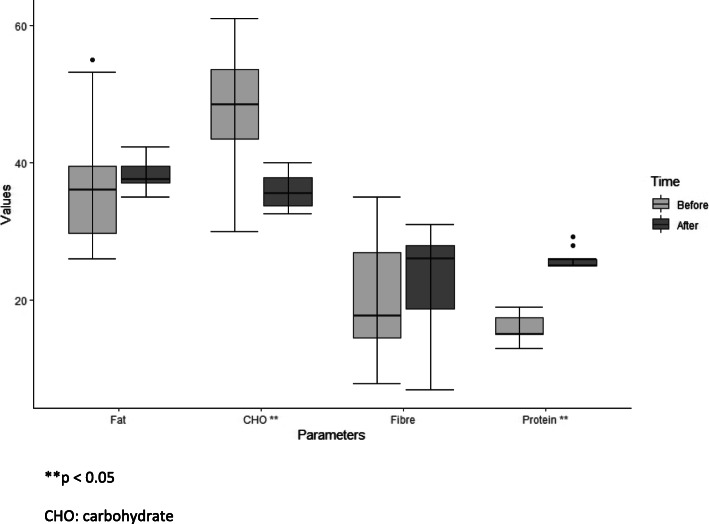
Diet composition before and after *exercise + diet* period. **p < 0.05. CHO: carbohydrate

The analysis of the dietary diaries showed a significant modification of the habitual diet during the *exercise + diet* period, with an increase in protein intake and a decrease in carbohydrate intake (Fig. 1). The percentage of calories introduced with proteins was 96%(median value) of those prescribed, and 145% of those introduced with the habitual diet (*p* < 0.05).

## Discussion

The main aim of the present study was to evaluate, in LOPD patients long term treated with ERT, the effects of exercise alone (*exercise*) or combined to a high-protein diet (*exercise + diet*) on indices of exercise tolerance, motor and respiratory functions and quality of life. A *control* period of no intervention was also present in the protocol.

Interestingly, we observed that the V̇O_2_ peak decreased in *control*, whereas it increased in *exercise* and more significantly so in *exercise + diet*. V̇O_2_ peak, a close estimate of maximal aerobic power, is traditionally considered an index evaluating the maximal performance of the integrated respiratory, cardiovascular and muscular factors governing oxidative metabolism during exercise, in other words the maximal flux of O_2_ from ambient air to the mitochondria of skeletal muscles [20]. In more functional terms, V̇O_2_ peak estimates the maximal power which can be sustained by oxidative metabolism, and therefore the maximal power which can be sustained for a relatively long period of time (several minutes) without incurring in fatigue. Besides its obvious relationship with exercise tolerance, and therefore with the patients’ quality of life, it has been demonstrated that in different pathological conditions (such as cardiovascular or respiratory diseases) V̇O_2_ peak also possesses a strong prognostic relevance [21]. It is noteworthy that during the 6-month period of *control* V̇O_2_ peak showed a significant decrease. This seems to be compatible with the clinical observation that many patients long term treated with ERT show disease progression despite the therapy [9, 10]. Our data suggest that regular exercise, in particular in association with a high-protein diet, can counteract the progressive loss of V̇O_2_ peak.

Rather surprisingly, the observed changes in V̇O_2_ peak were not associated with changes in the results obtained during the 6MWT, which did not change following any experimental conditions. This discrepancy further supports data from a previous study by our group [6], suggesting that in LOPD patients V̇O_2_ peak is a more sensitive parameter than the distance walked during 6MWT in the assessment of the patients’ exercise tolerance. It is also possible that the 6MWT performance is more related that the V̇O_2_ peak to the strength of the proximal muscle of the legs. Indeed, no changes in muscle strength were observed in all experimental conditions. This is presumably attributable to the very moderate resistance to movements imposed by the elastic bands. The very moderate resistance was chosen to avoid risks of inducing muscle damage.

The results in terms of V̇O_2_ peak are compatible with those found in relation to the quality of life evaluation, which showed a slight amelioration after *exercise*, and significant improvements for different aspects after *exercise + diet*. Interestingly, the association of the high-protein diet with exercise improved also other clinically relevant outcomes, such as the blood levels of muscular enzymes (which showed a decrease, especially of LDH), and variables of respiratory function (VC and FEV_1_); the improvements of blood levels of muscular enzymes and of spirometry variables were not observed after *exercise* alone. Based on these results, it can be hypothesized that high protein diet has an effect in restoring not only skeletal muscle function but also respiratory muscles.

The rationale to combine *exercise + diet* in LOPD patients comes from cell pathology. The damage of muscle fibers in these patients is indeed not only related to glycogen accumulation, but also to secondary derangements such as increased proteolysis and aberrant autophagy [22]. Exercise and diet could counteract this process, acting on different mechanisms. In fact, in muscle fibers aerobic exercise can modify energy metabolism, increasing the use of fatty acids as an alternative source of energy, thus reducing proteolysis, autophagy and muscle damage [17]*.* Moreover, exercise training can counteract the general deconditioning typical of chronic diseases, as well as the chronic inflammatory condition associated with inactivity [23]. On the other hand, the high-protein low-carbohydrate diet can reduce glycogen deposition in muscle fibers and increase intracellular protein synthesis, thereby reducing glycogen accumulation, proteolysis, muscle autophagy and damage [17, 24].

Before the approval of ERT, many clinicians prescribed diet and exercise to LOPD patients, based on data by Slonim et al. obtained in a 10 years retrospective study on 34 patients with LOPD, treated with the *so-called* NET (nutrition and exercise therapy) [11]. The nutritional approach adopted by Slonim et al. consisted of 25–30% protein, 30–35% carbohydrate, and 35–40% fat, which is the same approach used in the present study. Patients on NET also took supplements of the aminoacid L-alanine, which was not used in the present research since further studies failed to demonstrate the clinical efficacy of this supplementation [25]. In NET the exercise intervention consisted of a daily treadmill program for 45–50 min, followed by aerobic upper-body exercise for 10–15 min. In the present study we chose to use exercise on a cycle-ergometer to allow the participation of patients who had ambulatory difficulties and proposed only for 4 sessions a week in order to make the intervention more compatible with the patients’ everyday life and to increase their compliance. The rate of clinical deterioration (as estimated by the Walton Scale) during NET was compared with the rate of deterioration observed before starting NET: the data showed that the more compliant patients responded far better than those who became less active and did not follow the diet [11]. No differences were found for the Walton scale score in our study, probably since the *control* and intervention periods of 6 months were too short to observe modifications on this scale.

After the advent of ERT for Pompe disease, only a few studies investigated the effects of exercise on ERT-treated patients [12, 13, 26] and, to the best of our knowledge, no studies investigated the effects of a high-protein diet associated with exercise in these patients. The advantages of associating physical exercise with ERT were first demonstrated in a study performed in the mouse model of Pompe disease, in which the animals underwent treadmill training and showed improved aerobic capacity, strength and motor functions [27]. In 2011 Terzis et al. [12] reported the effects of a 20-week exercise training in 5 LOPD patients receiving ERT. The prescribed exercise consisted in 3 sessions a week, including 30 min of cycling, stretching and resistance training. Significant increases in muscular strength and in the distance walked during the 6MWT were observed after training [12]. More recently, Van der Berg et al. [13] performed a larger study, including 25 patients with LOPD who performed 12 weeks of exercise training while continuing their ERT infusions. The prescribed exercise consisted in 3 sessions a week of training combining aerobic, resistance and core stability exercises, utilizing a cycle ergometer and other gym instruments such as chest press, biceps curl, leg press and leg curl. After training V̇O_2_ peak and maximum workload capacity improved significantly, while pulmonary function (VC) did not change. Increases in strength of the hip flexors and the shoulder abductors were also observed. The distance walked during the 6MWT increased by 6%. Two patients experienced significant increases of CK but the exercise was generally well tolerated [13]. Compared to the study of Van der Berg et al. [13] our population of LOPD patients seems to be more severely affected at baseline, with 11 out of 14 patients having walking difficulties; this might explain the lack of significant changes in the 6MWT and in muscle strength in our patients after *exercise.* Overall, a comparison with previous studies carried out prescribing exercise in patients receiving chronic ERT seems to confirm the concept that exercise alone does not ameliorate blood levels of muscle enzymes (CK actually tends to increase) and variables of pulmonary function, which, can ameliorate when an high-protein diet is introduced in addition to exercise. (see Slonim et al. [11] and the present study).

The lack of previous studies on nutritional interventions in LOPD patients receiving chronic ERT could be linked to the difficulty of guaranteeing compliance to high-protein diets, as a consequence of the lower palatability of many high-protein foods compared to those with high carbohydrate content, or of the higher satiety power of proteins, which may lead to a reduction in food intake [28]. A review [29] published in the pre-ERT era, dealing with LOPD patients on high-protein diets, reported that only 25% of patients showed improvements in muscle or respiratory function. However, the compliance to the diet in most of the studies mentioned in that review was very poor. The authors attributed the problems of compliance to the large amount of prescribed proteins and to the associated perceived risk of weight gain [29]. Conversely, in our study we obtained a very high compliance to the prescribed diet, presumably as a consequence of the personalization of the food composition of the diet carried out by experienced nutritionists who took in consideration the patients’ preferences and habits. A strict contact between patients and dieticians was assured throughout the *exercise + diet* intervention. The risk of weight gain was not confirmed by our data, which on the contrary showed a significant reduction of BMI during *exercise + diet*, even in patients following normocaloric diets. A high-protein diet, as the one prescribed in the present study, should be carefully prescribed and monitored in underweight patients, and the different dietary sources of proteins should be wisely chosen, assuring great variety between meat, fish, eggs, dairy products and vegetables proteins (as beans or nuts). Some experimental and observational human studies have suggested that a highprotein intake may increase kidney diseases, but only a few randomized trials with an observation time longer than 6 months have been carried out on this topic; most of these studies, moreover, were conducted in patients with pre-existing diseases that predispose them to the development of kidney diseases [30]. In any case, a screening for kidney diseases and a strict monitoring of creatinine and urinary parameters should be considered before and during a long-term treatment with a high-protein diet. Of note, no changes in creatinine plasma values were observed throughout the study.

The present study presents several limitations. In particular, the total number of patients who completed the 3 periods was relatively limited. This could be explained, at least in part, by considering the rarity of the disease and the length of the study (overall duration 24 months). Despite the relatively low number of patients, however, the obtained data showed statistically significant differences in some of the main variables of interest, following some of the proposed interventions. This could be attributed to the elevated compliance by the patients to the proposed life style interventions, facilitated by the constant motivational contacts between researchers and patients. Eight out of the 10 patients who completed the *exercise + diet* period were keen on following the same prescriptions also after the end of the study. Long term follow-up data of these patients are necessary to see if the compliance and the beneficial effects of the interventions will be maintained.

## Conclusion

In this study a high-protein diet associated with moderate-intensity aerobic exercise improved exercise tolerance, muscle enzymes, pulmonary function and quality of life of LOPD patients chronically treated with ERT. Since LOPD in advanced stages causes significant disability and ERT efficacy seems to decrease during long-term treatment, moderate-intensity aerobic exercise training in association with a high-protein diet might be promoted as part of the standard care of these patients, in order to reduce their demand for assistance and improve their quality of life.

## Data Availability

Data are stored and available at the center according to the national laws.

## Abbreviations

GAA: Acid alpha glucosidase
LOPD: Late onset Pompe Disease
ERT: Enzyme replacement therapy
HR: Heart rate
BMI: Body mass index
ECG: Electrocardiography
CWR: Constant work rate
VE: Pulmonary ventilation
VT: Tidal volume
FR: Respiratory frequency
VO2: O_2_ uptake
VCO2: CO_2_ output
R: Gas exchange ratio
SpO2: Arterial oxygen saturation
RPE: Ratings of perceived exertion
CK: Creatine phosphokinase
LDH: Lactate dehydrogenase
ALT: Alanine transaminase
AST: Aspartate transaminase
VC: Vital capacity
FEV1: Forced expiratory volume
6MWT: 6-min walking test
